# Comparative Lipid Profiling of the Cnidarian *Aiptasia pallida* and Its Dinoflagellate Symbiont

**DOI:** 10.1371/journal.pone.0057975

**Published:** 2013-03-04

**Authors:** Teresa A. Garrett, John L. Schmeitzel, Joshua A. Klein, Janice J. Hwang, Jodi A. Schwarz

**Affiliations:** 1 Department of Chemistry, Vassar College, Poughkeepsie, New York, United States of America; 2 Department of Biology, Vassar College, Poughkeepsie, New York, United States of America; 3 Department of Computer Science, Vassar College, Poughkeepsie, New York, United States of America; CNRS, France

## Abstract

Corals and other cnidarians house photosynthetic dinoflagellate symbionts within membrane-bound compartments inside gastrodermal cells. Nutritional interchanges between the partners produce carbohydrates and lipids for metabolism, growth, energy stores, and cellular structures. Although lipids play a central role in the both the energetics and the structural/morphological features of the symbiosis, previous research has primarily focused on the fatty acid and neutral lipid composition of the host and symbiont. In this study we conducted a mass spectrometry-based survey of the lipidomic changes associated with symbiosis in the sea anemone *Aiptasia pallida*, an important model system for coral symbiosis. Lipid extracts from *A. pallida* in and out of symbiosis with its symbiont *Symbiodinium* were prepared and analyzed using negative-ion electrospray ionization quadrupole time-of-flight mass spectrometry. Through this analysis we have identified, by exact mass and collision-induced dissociation mass spectrometry (MS/MS), several classes of glycerophospholipids in *A. pallida*. Several molecular species of di-acyl phosphatidylinositol and phosphatidylserine as well as 1-alkyl, 2-acyl phosphatidylethanolamine (PE) and phosphatidycholine were identified. The 1-alkyl, 2-acyl PEs are acid sensitive suggestive that they are plasmalogen PEs possessing a double bond at the 1-position of the alkyl linked chain. In addition, we identified several molecular species of phosphonosphingolipids called ceramide aminoethylphosphonates in anemone lipid extracts by the release of a characteristic negative product ion at *m/z* 124.014 during MS/MS analysis. Sulfoquinovosyldiacylglycerol (SQDG), an anionic lipid often found in photosynthetic organisms, was identified as a prominent component of *Symbiodinium* lipid extracts. A comparison of anemone lipid profiles revealed a subset of lipids that show dramatic differences in abundance when anemones are in the symbiotic state as compared to the non-symbiotic state. The data generated in this analysis will serve as a resource to further investigate the role of lipids in symbiosis between *Symbiodinium* and *A. pallida*.

## Introduction

**Figure 1:**
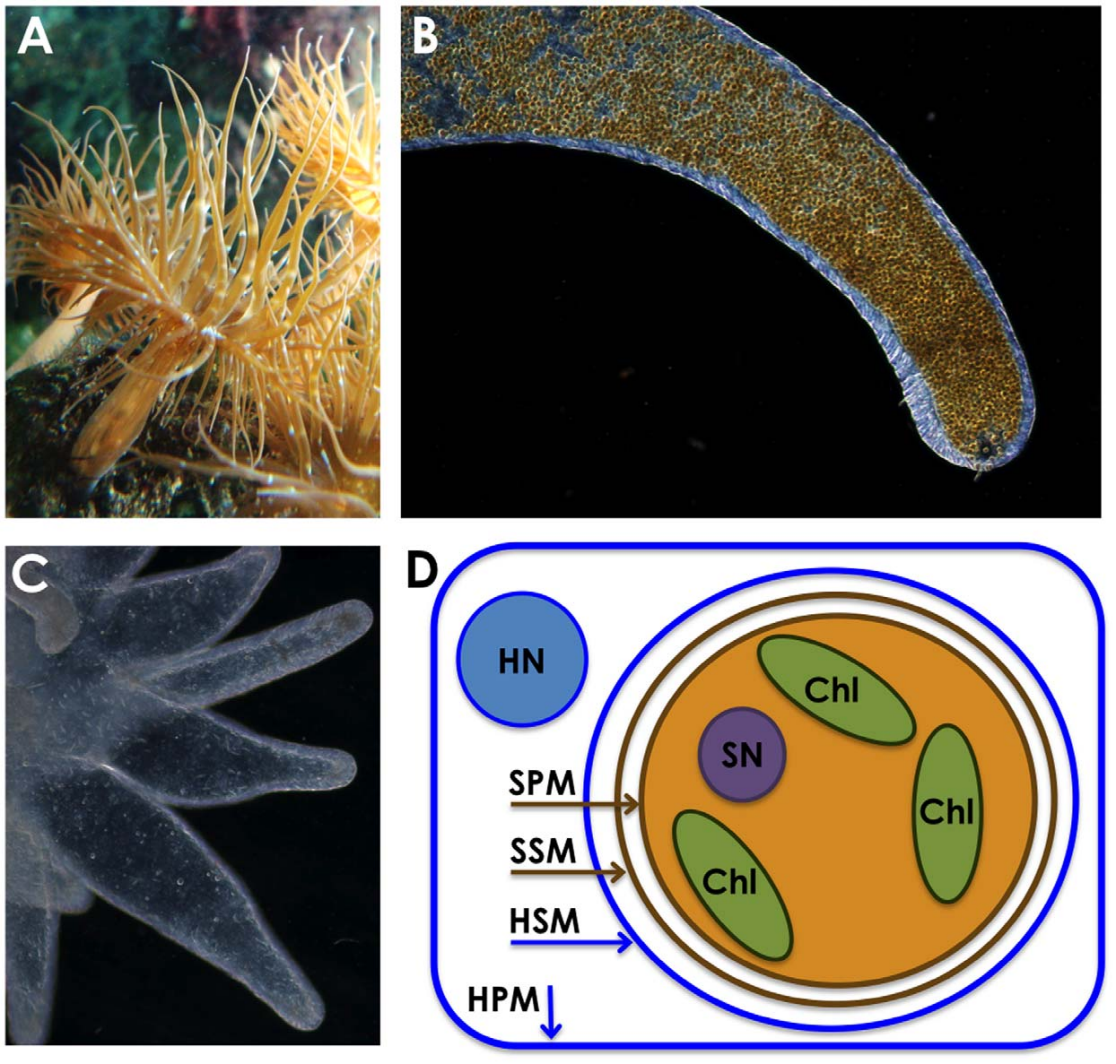
The symbiosis between the sea anemone *Aiptasia pallida* and the dinoflagellate *Symbiodinium*. Panel A: Symbiotic polyps of *Aiptasia pallida* demonstrate a golden brown color conferred on them by photosynthetic pigments from *Symbiodinium*. Panel B: A single tentacle from a symbiotic polyp contains thousands of individual *Symbiodinium* cells visible as small golden brown spheres within the gastrodermal tissue of the tentacle (epidermal tissue does not contain symbionts and is visible as the thin transparent layer at the periphery of the tentacle). Panel C: Four tentacles from an aposymbiotic polyp demonstrate the absence of *Symbiodinium* in the gastrodermis. Panel D: A schematic diagram of the intracellular arrangement of membranes of the host (blue) and symbiont (brown). The symbiont lives enclosed in the symbiosome, a membrane-bound compartment that arises from the host phagosome but that likely is modified upon establishment. HN = host nucleus, SN = symbiont nucleus, Chl = symbiont chloroplasts, HPM = plasma membrane of the host cell, SPM = plasma membrane of the symbiont cell, SSM = putative symbiosome membrane contributed by the symbiont shedding of external membrane, HSM = symbiosome membrane that arises from the phagosome membrane produced when the host takes up the symbiont into the host cell via phagocytosis.

The coral reef ecosystem is entirely dependent upon a symbiosis between photosynthetic dinoflagellates and coral. The dinoflagellate symbionts live inside cells of coral and other cnidarian hosts, where they supply photosynthate that fuels the metabolism, growth, reproduction, and calcification of the host [1], [2]. In return, the symbionts’ growth is supported by conservation and recycling of nutrients supplied in part through the metabolic waste products of the host [3], [4]. The density of symbionts can be quite high in the tentacles and oral disk of the host, exceeding 10^6^ symbiont cells per mg of host tissue and conferring a golden brown color on their host’s otherwise transparent tissue (Figure 1). Under conditions of environmental stress, in particular increases in temperature, the symbiosis fails, and symbionts are jettisoned from the host or are killed [5], [6]. The loss of symbionts has been termed “coral bleaching” and is correlated with dramatic declines in coral growth and health, and there is considerable concern that climate change-associated fluctuations in temperature has led to the large scale degradation of reef ecosystems [7].

For most species of cnidarians that host symbiotic dinoflagellates, the host must acquire symbionts from the environment, incorporate them into their gastrodermal cells, regulate growth of both symbiont and host cells, and manage the interchange of materials and molecules. All of these processes operate at the cellular and molecular level. Therefore, it is critical that we understand the molecular and cellular processes that give rise to symbiosis and that are involved in regulating and maintaining the symbiosis.

Despite the significance of cnidarian symbiosis, we are still in the early stages of understanding the symbiosis at a cellular and molecular level. In recent years, there has been an active body of research that has begun investigating the genomic and proteomic characteristics of the symbiosis and its breakdown [2], [8], and it is clear that host and symbiont undergo a complex series of molecular-level communications to establish and engage in symbiosis. However, there is not yet an understanding of the role of lipids in the symbiosis despite their obvious role in symbiosis.

The symbiotic relationship in cnidarians is typically established upon phagocytic engulfment of the dinoflagellate symbiont by individual cells of the cnidarian gastroderm [9], [10]; the resulting phagosome fails to fuse with lysosomes [9], [11], [12]. Structurally, the establishment of symbiosis is accompanied by the formation of a novel membrane-bound compartment, the symbiosome, that arises from the phagosome, and which encloses the symbiont within the host cell (Figure 1). It is unknown to what extent the establishment of this membrane is accompanied by lipid biosynthesis. In many species of symbiotic cnidarians, the membrane-delimited symbiosome compartment occupies a substantial volume of the host cell, suggesting that the uptake of symbionts must be accompanied by substantial regeneration of the plasma membrane and endomembrane system. It is not known the extent to which the phagosome membrane incorporates new lipids that become synthesized upon the onset of symbiosis or by donation of lipids from the symbiont. While the protein composition of this membrane has been a subject of investigation [11], [13], there is little information about the lipid composition of the symbiosome membrane [12], [14].

In addition to the creation of a novel organelle that is established by the symbiosis, the flux of metabolites between the partners must be accompanied by a significant amount of lipid biosynthesis, yet there is almost no information about the underlying lipid composition of either the host or symbiont when they are living outside of symbiosis or when they are living together in a symbiotic union. The information that is available focuses primarily on fatty acid composition and not the type of lipid to which the acyl chains might be attached [15], [16]. Neutral lipid stores [15], localized in part to lipid bodies [17], [18], have also been investigated previously. Furthermore, it has yet to be determined how the lipid profile changes when host cells become infected with symbionts, or when symbionts begin producing metabolites that might be used in lipid synthesis or catabolism.

*Aiptasia*, a tropical sea anemone that is broadly distributed globally across tropical shallow marine environments, is a model system in which to investigate cellular and molecular questions of coral symbiosis [19], [20]. Like corals, *Aiptasia* hosts *Symbiodinium* cells within their gastroderm. The experimental advantage of this species is that the anemone host and the dinoflagellate symbiont *Symbiodinium* can be separated from each other and cultured independently, facilitating the identification of symbiosis-related molecules through comparisons of the symbiotic and non-symbiotic condition of both the host and symbiont.

In most of its geographical range, *Aiptasia pallida* hosts clade B *Symbiodinium,* but uniquely in the Florida Keys, *Aiptasia* primarily hosts a clade A *Symbiodinium*, and can also occur in mixed clade A/clade B infections [21], [22]. Isolates of both clade A and B isolates have been cultured from Florida Keys *Aiptasia* and are maintained in culture [22].

Using a mass spectrometry-based approach we have investigated the lipid composition of the anemone *A. pallida* and its symbiont, clade A *Symbiodinium.* Through this work we identified several lipids found in *A. pallida* and *Symbiodinium* and have begun to determine the lipids that are altered when the partners are in symbiosis. This work is foundational for determining the specific lipid metabolic changes during different stages of symbiosis and necessary to begin to identify the biochemical processes involved in this essential interaction between symbiont and host.

## Materials and Methods

### Materials

Reagent and HPLC-grade chloroform, methanol and water were from Sigma-Aldrich. All other reagents were from Sigma-Aldrich or VWR.

### Growth and Sampling of *Aiptasia pallida*

All anemones were derived from the clonal line CC7 [20], [23] and were cultured in a 12 h:12 h light:dark regime at 25°C and fed 3× per week with freshly hatched *Artemia* (brine shrimp). Aposymbiotic anemones were derived from symbiotic anemones by cultivation in the dark in the herbicide Diuron for 1 month, followed by a 3 month recuperation period in *Symbiodinium-*free artificial seawater (ASW) in the same light cycle and feeding regime as the symbiotic anemones. Aposymbiotic anemones were checked regularly using fluorescence microscopy to ensure they were free of *Symbiodinium.* Biological replicates were established by collecting a sample of anemones at noon every other day for a total of three replicate samples. All anemones were starved for three days prior to sampling to avoid contamination by lipids of their brine shrimp prey.

### Growth and Sampling of *Symbiodinium*

*Symbiodinium* FLAp1AB, a clade A isolate, was originally isolated from Florida Keys *Aiptasia* [22]. The cells were cultured in F/2 media in ASW in a 12 h:12 h light:dark cycle at 25°C. Cultures were harvested 1 week after inoculation of fresh culture media, during active growth phase.

### Lipid Extract Preparation

Cultures of *Symbiodinium* FLAp1AB (400 mL) were harvested by centrifugation at 2000×g for 20 minutes at 4°C. The harvested cells were re-suspended in 1.2 mL of phosphate buffered saline (PBS; 137 mM NaCl, 2.7 mM KCl, 10 mM Na_2_HPO_4_, 1.8 mM KH_2_PO_4_). *A. pallida*, living either symbiotically or aposymbiotically (∼0.1 g wet weight) were homogenized in 1.2 mL of PBS. After homogenization, each sample was divided equally into three glass tubes equipped with Teflon-lined lids to generate technical triplicates. Chloroform (0.5 mL) and methanol (1.0 mL) were added to each to generate a single-phase neutral Bligh-Dyer extraction mixture (1∶2∶0.8, CHCl_3_:CH_3_OH:PBS, v:v:v) [24]. After vigorous mixing the extraction mixture was incubated at room temperature for 5 minutes and then centrifuged for 5 minutes in a clinical centrifuge to remove the cell debris. The supernatant was transferred to a fresh glass tube and 0.5 mL CHCl_3_ and 0.5 mL PBS were added to generate a two-phase neutral Bligh-Dyer extraction mixture (2∶2∶1.8, CHCl_3_:CH_3_OH:PBS, v:v:v). The phases were resolved by centrifugation. The upper phase and any interphase material was discarded. The lower phase was washed with 1.9 mL of pre-equilibrated neutral lower phase, the phases resolved by centrifugation, and the resulting lower phase transferred to a fresh tube and dried under N_2_.

### Acid Hydrolysis of Total Lipid Extracts

Total lipid extracts from *A. pallida* in symbiosis with *Symbiodinium* FLAp1AB were subjected to acid hydrolysis as described in Zemski Berry and Murphy [25].

### Mass Spectrometry of Total Lipid Extracts

The dried lipid film was re-dissolved in 200 µl of CHCl_3_:CH_3_OH (2∶1, v:v). This solution was directly infused into the turbo electrospray ionization source of a QSTAR XL quadrupole time-of-flight tandem mass spectrometer (ABI/MDS-Sciex, Toronto, Canada) at 6 µl/min detecting in the negative-ion mode as described previously [26].

### Liquid Chromatography Electrospray Ionization Mass Spectrometry

Dried lipid films were re-dissolved in 200 µL of CHCl_3_:CH_3_OH (2∶1, v:v). The samples were separated using normal phase chromatography as described previously [26]. The column effluent, flowing at 0.4 mL/min, was infused into an Agilent 6520 quadrupole time-of-flight mass spectrometer. Samples were ionized by electrospray ionization and ionized in both the positive or negative mode as indicated. Mass spectra were obtained scanning from *m/z* 100 to 2000 at 1 spectra per second. The instrument parameters were as follows: fragmentor voltage –175 V, drying gas temperature –325°C, drying gas flow –11 L/min, nebulizer pressure –45 psig, capillary voltage –4000 V. Data were collected in profile mode with the instrument set to the 3200 mass range under high resolution conditions at 4 GHz data acquisition rate. For MS/MS analysis, spectra were obtained scanning from *m/z* 100 to 2000 at 1 spectra per second with an isolation width of ∼4 *m/z*. The collision energy was −40 (laboratory frame of reference) and N_2_ was the collision gas. The instrument was calibrated using Agilent ESI-L low concentration tuning mix. Under normal operating conditions the resolution of the instrument was ∼15,000. The mass accuracy was between 1 and 5 ppm, therefore measured masses are given to three decimal places. Data were acquired using Agilent MassHunter Workstation Acquisition Software and analyzed using MassHunter Workstation Qualitative Analysis Software (Agilent Technologies, Santa Clara, CA). Exact mass measurements were obtained using CS ChemBioDraw Ultra 12.0.

### Data Analysis

Negative-ion LC-MS data was aligned, normalized and compared using the Scripps Center for Metabolomics XCMS online resource [27], [28]. Nine replicate datasets (consisting of three technical replicates for each of the three biological replicates) were loaded to the XCMS Online server and analyzed using parameters optimized for HPLC-QTOF data acquired in the negative mode. XCMS generated the average *m/z*, average retention time, and average intensity of the replicate LC-MS data sets. To identify differences in lipid abundance between the symbiotic states, the mean of three technical replicates for each of three symbiotic anemones and three aposymbiotic anemones were compared by Students t-test followed by correction for false discovery rate using the Benjamini-Hochberg method with a false discovery rate of 0.01 (1%) [29]. This produced 1539 features that met the FDR cut-off (at p = 8.31×10^−4^). The log fold change, retention time, and *m/z* were plotted using MatLab.

## Results

### Initial Profiling of Total Lipid Extracts from *A. pallida* and *Symbiodinium* FLAp1AB Using Negative-ion Mass Spectrometry

**Figure 2:**
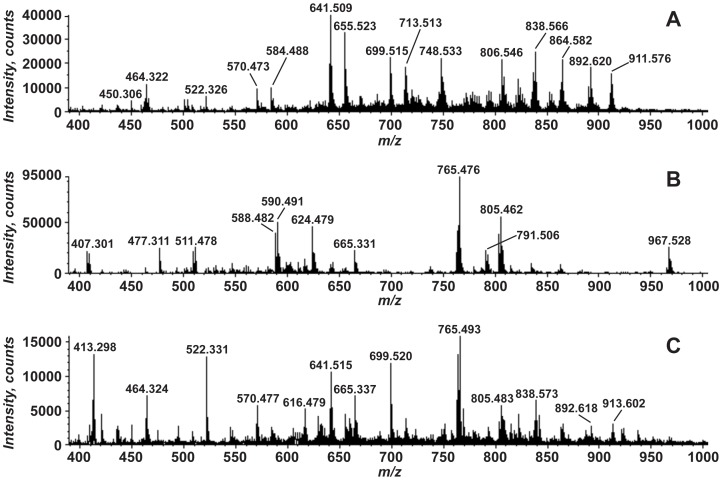
Negative-ion electrospray ionization mass spectrometry of total lipid extracts from *A. pallida* and *Symbiodinium*. Total lipid extracts from aposymbiotic *A. pallida* (Panel A), *Symbiodinium* FLAp1AB (Panel B), and *A. pallida* in symbiosis with *Symbiodinium* (Panel C) were analyzed by direct infusion into a quadrupole time-of-flight mass spectrometer and analyzed in the negative mode. The spectra from *m/z* 400–1000 are shown.

Total lipid extracts prepared from non-symbiotic anemones (aposymbiotic anemones), symbiotic anemones and non-symbiotic (cultured) *Symbiodinium* FLAp1AB were analyzed using negative-ion electrospray ionization quadrupole time-of-flight mass spectrometry (MS). Figure 2 shows the total lipid profiles of each from *m/z* 400 to 1000. We clearly observe differences among these spectra that suggest that the lipidome of the symbiotic anemone may not be simply the sum of the anemone and symbiont lipidomes.

### Glycerophospholipids Identified in *A. pallida*

**Figure 3:**
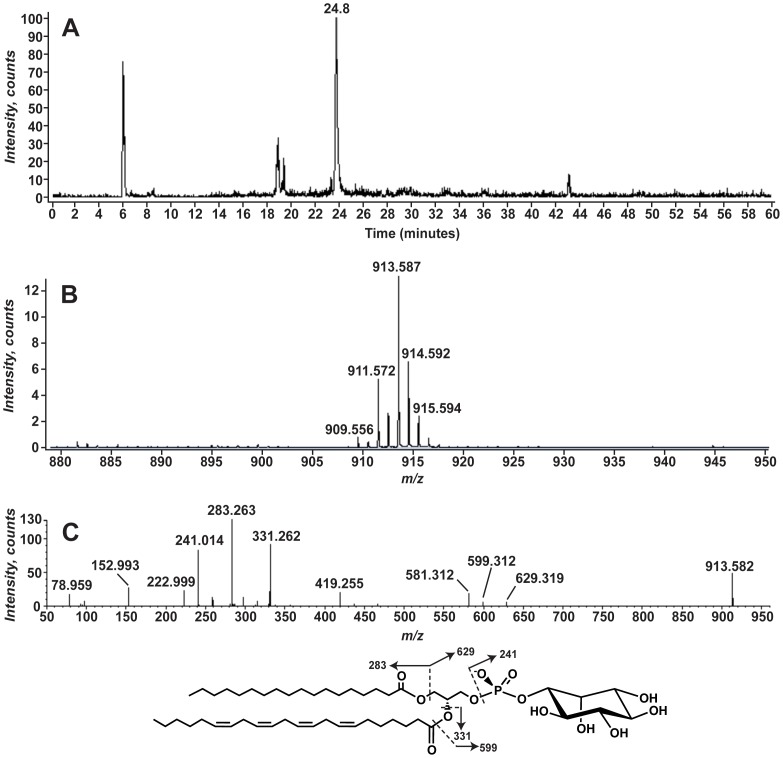
Phosphatidylinositol identified in aposymbiotic *A. pallida* total lipid extracts. The total lipid extracts from aposymbiotic *A. pallida* were analyzed using normal phase liquid chromatography mass spectrometry (LC-MS) detecting negative ions. The extracted ion current (EIC) for *m/z* 913.5, the mass of 40∶4 PI is shown in Panel A. Panel B shows the mass spectrum from *m/z* 880 to 950 for the lipids eluting between 23.6 and 24.0 minutes. The MS/MS spectrum of *m/z* 913.5 is shown in Panel C. The structure of the predominant PI molecular species and the predicted product ions is shown below panel C.

From this analysis we also identified several typical eukaryotic glycerophospholipids (GPLs) in aposymbiotic *A. pallida*. Total lipid extracts were analyzed using normal phase liquid chromatography electrospray ionization mass spectrometry (LC-MS). The predominant ions between *m/z* 880 and 950, that eluted at a retention time of ∼23.8 minutes, were identified as singly-charged negative ions ([M-H]^−^) of phosphatidylinositol (PI) by exact mass and collision-induced dissociation mass spectrometry (MS/MS) analysis (Figure 3 and Table 1). The predominant species correspond to PI with 40 total carbons and 4 unsaturations in the acyl chains (40∶4 PI). Figure 3C shows the MS/MS results for the ion at *m/z* 913.582. The product ions at 78.959 and 152.993 correspond to PO_3_^−^ and C_3_H_6_O_5_P^−^ (exact mass 78.9591 and 152.9958, respectively) consistent with a GPL (Figure 2C) [30], [31]. Acyl chains corresponding to 18∶0 (*m/z* 283.263, exact mass 283.2637) and 22∶4 (*m/z* 331.262, exact mass 331.2637) are also seen. The product ion at *m/z* 241.014 corresponds to phosphoinositol head group (C_6_H_10_O_8_P^−^, exact mass: 241.0119) [31], [32]. MS/MS of the other PI species, such as at *m/z* 911.572 and 909.556, yielded similar product ion spectra, particularly the formation of the product ion at *m/z* 241.01. The differences in the product ion spectra are attributable to the differences in acyl chain composition among the different PIs. Table 1 shows the variety of PI molecular species detected in the aposymbiotic anemone lipid extracts and the observed acyl chains for each, inferred from the exact mass and MS/MS analysis.

**Table 1:** Phosphatidylinositol species identified in *A. pallida* lipid extracts.

[M-H]^−^	Acyl ChainComposition*	Acyl Chain Combinations Observed#
883.5	39∶5	17∶1, 22∶5
885.5	39∶4	17∶1, 22∶4
909.5	40∶6	18∶1, 22∶5
911.5	40∶5	18∶0, 22∶5
		18∶1, 22∶4
913.5	40∶4	18∶0, 22∶4
919.5	41∶8	19∶1, 22∶7
921.5	41∶7	19∶1, 22∶6
923.5	41∶6	19∶1, 22∶5
925.5	41∶5	19∶1, 22∶4

* Total number of carbons:total unsaturations.

# The position of the acyl chain cannot be determined from this data.

**Figure 4:**
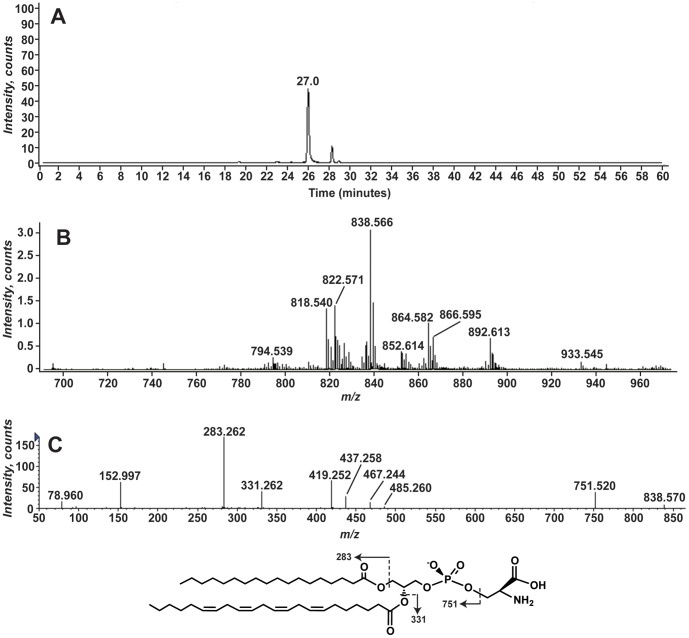
Phosphatidylserines identified in aposymbiotic *A. pallida* total lipid extracts. *A. pallida* total lipid extracts were analyzed as described in the legend for Figure 2. Panel A shows the EIC for *m/z* 838.5. Panel B shows the mass spectrum from *m/z* 700 to 980 for the lipids eluting between 25.6 and 26.4 minutes. The MS/MS spectrum of *m/z* 838.5 is shown in Panel C. The structure of the 40∶4 PS is shown below with the predicted product ions.

Similarly, we identified a series of phosphatidylserine (PS) molecular species (Figure 4). LC-MS analysis revealed [M-H]^−^ ions at *m/z* 700 to 960 that eluted at a retention time of ∼26 minutes (Figure 4A). MS/MS analysis of the predominant ion at *m/z* 838.566 is consistent with PS (Figure 4C). In particular, the product ion at *m/z* 751.520 corresponds to the neutral loss of 87 a.m.u and is consistent with the loss of the serine headgroup [33]. Similar to PI, product ions typically observed in GPLs are also observed at *m/z* 152.997 and 78.960 [30], [32], [33]. Product ions at *m/z* 283.262 and 331.262 correspond to 18∶0 and 22∶4 fatty acids. In addition, product ions corresponding to the loss of the serine head group and the 18∶0 or 22∶4 fatty acids as either the free acid (RCOOH) or as a ketene (RCH = CO) are detected in between *m/z* 400 and 500. The loss of the 18∶0 acyl chain and head group yields product ions at *m/z* 485.260 and 467.244. The analogous loss of the 22∶4 acyl chain yields the product ions at *m/z* 437.271 and 419.262. Several different PS molecular species with the acyl chain compositions shown in Table 2 are detected. The MS/MS spectra all display the product ions consistent with a GPL, a neutral loss of 87, and acyl chains consistent with their exact mass.

**Table 2:** Phosphatidylserine species identified in *A. pallida* lipid extracts.

[M-H]^−^	Acyl ChainComposition*	Acyl Chain Combinations Observed#
818.5	39∶7	17∶1, 22∶6
820.5	39∶6	17∶1, 22∶5
822.5	39∶5	17∶1, 22∶4
834.5	40∶6	18∶0, 22∶6
		18∶1, 22∶5
		20∶5, 20∶5
836.5	40∶5	18∶0, 22∶5
		18∶1, 22∶4
		20∶4, 20∶4
838.5	40∶4	18∶0, 22∶4
850.5	41∶5	19∶1, 22∶4
852.5	41∶4	19∶0, 22∶4
862.5	42∶6	20∶1, 22∶5
		20∶2, 22∶4
864.5	42∶5	20∶1, 22∶4
866.5	42∶4	18∶0, 22∶4
890.5	44∶5	22∶2, 22∶4
892.5	44∶4	22∶4, 22∶4

* Total number of carbons:total unsaturations.

# The position of the acyl chain cannot be determined from this data.

**Figure 5:**
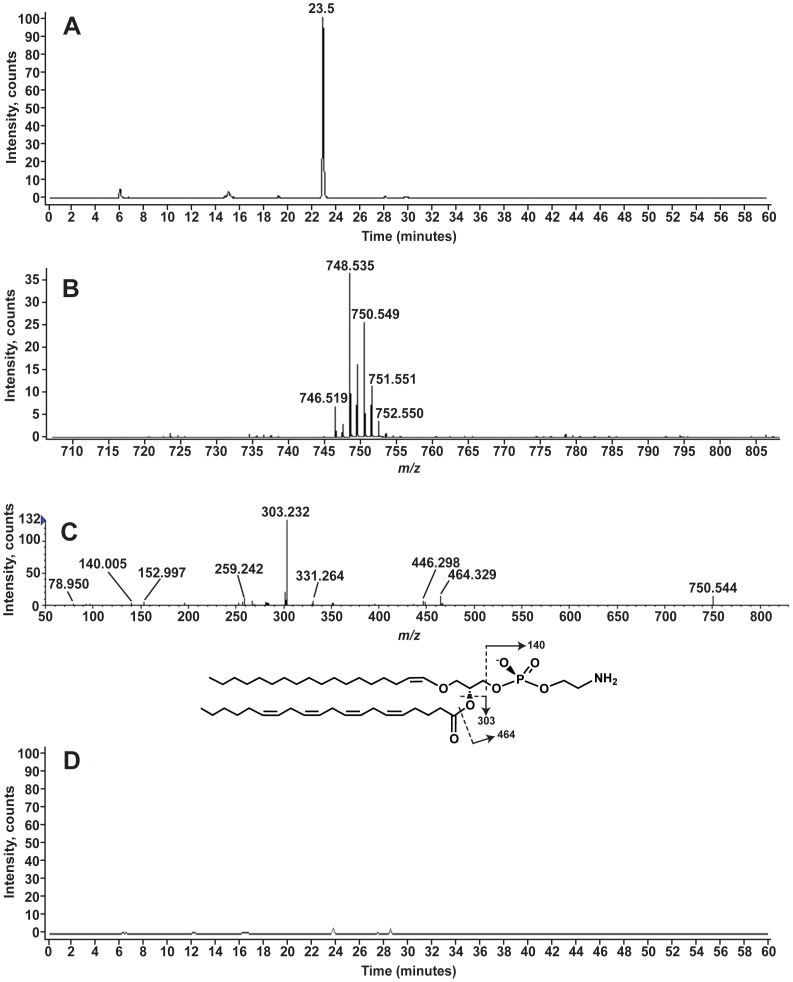
Plasmenyl phosphatidylethanolamines identified in *A. pallida*. Panel A shows the EIC of *m/z* 750.5, the mass corresponding to 38∶6 alkylPE. The negative-ion mass spectrum from *m/z* 700 to 810 for the lipids eluting between 22.8 and 23.5 minutes is shown in Panel B. Panel C shows the MS/MS analysis of 750.5. Panel D shows the EIC for *m/z* 750.5 following acid hydrolysis of the total lipid extract. The predicted structure is shown with selected product ions shown. The alkyl PE is shown with the double bond at the 1 position of the ether-linked chain as is known to occur for plasmalogen PE [25]. The product ion at *m/z* 331.264 suggests that a portion of the lipids at *m/z* 750.5 has a 22∶4 ester linked acyl chain and a 16-carbon ether linked chain.

 [M-H]^−^ ions suggestive of phosphatidylethanolamine (PE) were observed at a retention time ∼23 minutes during LC-MS analysis (Figure 5A). When the [M-H]^−^ ion at *m/z* 750.544 was analyzed by MS/MS, the product ions at *m/z* 78.950 and 152.997, consistent with a GPL [30], [32] and as shown above for PI and PS. The product ion at *m/z* 140.005 (Figure 5C) corresponds to the phosphoethanolamine head group (C_2_H_7_NO_4_^−^, exact mass 140.0018) are consistent with PE [25], [32]. However, the acyl chains observed and the exact mass of the precursor ion did not match PE with two ester linked acyl chains. In the MS/MS analysis of the ion at *m/z* 750.544, the product ions at *m/z* 303.232 and 331.264 indicated the presence 20∶4 and 22∶4 acyl chains. If both of these acyl chains were ester linked, the total mass would be 786.5079. The product ion spectrum is, however, consistent with a PE species that has an ether linked acyl chain, a 1-O-alkyl-2-acyl-glycerophosphoethanolamine [25], [34]. The product ion at *m/z* 464.329 corresponds to loss of a 20∶4 acyl chain as a ketene (RCH = CO) only if a 18 carbon chain with the equivalent of one unsaturation is attached to the glycerol as an ether. The product ion at *m/z* 446.298 corresponds to the loss of the 20∶4 acyl chain as a free fatty acid (RCOOH).

The location of the double bond cannot be definitively determined from this analysis. However, if the double bond is at the 1′ position of ether linked chain, then the resulting vinyl ether bond is susceptible to acid hydrolysis. To determine if the linkage was via an alkyl ether or alkenyl bond total lipid extracts from symbiotic anemones were subjected to acid hydrolysis and then analyzed by LC-MS. Figure 5D shows the extracted ion current (EIC) for the ion at *m/z* 750.5 for the LC-MS analysis of acid hydrolyzed total lipid extracts from symbiotic anemones. No significant amounts of ions corresponding to this or other PE molecular species were detectable following acid hydrolysis. In addition, the product ion at *m/z* 464.329 is more consistent, by mass, with the vinyl ether linkage than an acyl linkage (exact mass vinyl ether, 464.3156 versus acyl, 464.2783). In Figure 5C, therefore, we have depicted the linkage as a vinyl ether, consistent with plasmalogen PE (pPE) [25].

Several pPEs were detected (Figure 5B) in the aposymbiotic anemone lipid extracts. The acyl chain, released as a free acid during MS/MS analysis, is indicated in Table 3. For several of the pPEs, there are multiple acyl chains indicated. For those lipids, the alkyl linked acyl chain is varied such that the total number of carbons and unsaturations is maintained as indicated.

**Table 3:** Plasmalogen phosphatidylethanolamine species identified in *A. pallida* lipid extracts.

[M-H]^−^	Acyl Chain Composition*	Acyl Chains Observed#
720.5	36∶6	16∶1, 18∶1, 20∶5
722.5	36∶5	16∶0, 18∶1, 20∶4
734.5	37∶6	16∶1, 16∶0, 17∶1, 18∶2, 18∶1, 20∶5, 20∶4
736.5	37∶5	16∶1, 17∶1, 17∶0, 20∶4, 22∶4,
746.5	38∶8	16∶0, 17∶1, 18∶1, 18∶0, 20∶5, 22∶7, 22∶6, 22∶4
748.5	38∶7	16∶0, 17∶1, 18∶1, 18∶0, 20∶5, 20∶4, 22∶5, 22∶4
750.5	38∶6	18∶0, 20∶5, 20∶4, 22∶4
762.5	39∶7	18∶0, 20∶5, 20∶4, 22∶4
764.5	39∶6	16∶1, 18∶0, 20∶4, 22∶4
766.5	39∶5	16∶1, 17∶1, 17∶0, 18∶0, 20∶4, 22∶4
768.5	39∶4	16∶1, 16∶0, 17∶1, 17∶0, 18∶2, 18∶0
770.5	39∶3	16∶1, 16∶0, 17∶1, 17∶0, 18∶2, 18∶1, 18∶0,
772.5	39∶2	16∶1, 16∶0, 17∶1, 17∶0, 18∶2, 18∶1, 18∶0,
778.5	40∶6	17∶1, 18∶2, 18∶1, 20∶5, 20∶4, 20∶1, 20∶0, 22∶5, 22∶4
780.5	40∶5	16∶1, 18∶1, 20∶5, 20∶4, 20∶0, 22∶4
782.5	40∶4	16∶0, 17∶1, 20∶5, 20∶1, 22∶6,
792.5	41∶6	16∶1, 17∶1, 18∶1, 18∶0, 20∶5, 20∶4, 22∶4
794.5	41∶5	16∶0, 17∶1, 18∶0, 18∶1, 22∶4, 20∶4, 19∶2

* Total number of carbons:total unsaturations.

# This is likely the ester linked acyl chain. The second chain, attached via an ether linkage would not be observed in the MS/MS spectra.

**Figure 6:**
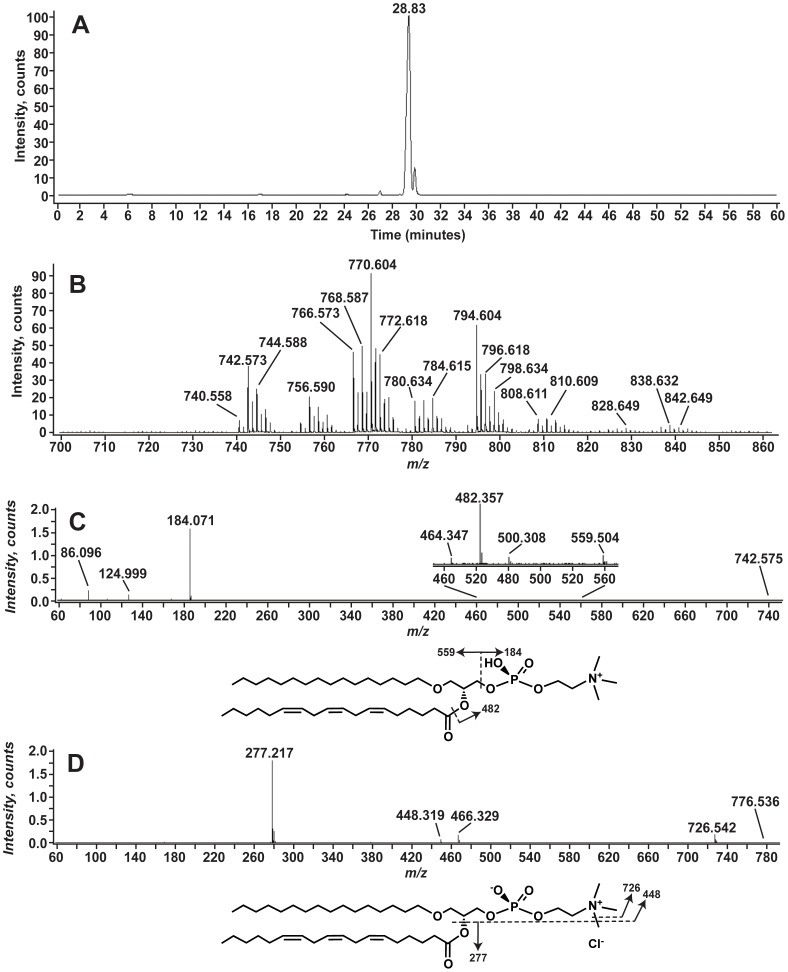
Alkyl-phosphatidylcholines identified in *A. pallida*. Panel A shows the EIC of the [M+H]^+^ ion at *m/z* 770.6. Panel B shows the positive-ion mass spectrum from *m/z* 700 to 860 for the lipids eluting between 28.9 and 30.1 Minutes. Panel C shows the positive-ion MS/MS spectrum. The aPC predicted from this spectrum and the exact mass is shown with selected product ions indicated. Panel D shows the negative-ion MS/MS spectrum with the major product ions are indicated on the aPC structure shown below.

When aposymbiotic anemone lipid extracts were analyzed using positive-ion LC-MS we detected a series of [M+H]^+^ ions that correspond to phosphatidylcholine (PC) molecular species. As shown in Figure 6A, these lipids eluted at a retention time of ∼29 minutes. MS/MS analysis of the ion at *m/z* 742.5 (Figure 6C) yields a product ion at *m/z* 184.071 that corresponds to the phosphocholine headgroup (C_5_H_15_NO_4_P^+^, exact mass 184.0733) [32]. The loss of the phosphocholine head group yields a minor product ion at *m/z* 599.504. The product ions at *m/z* 500.308, 482.357, and 464.347 correspond to loss of acyl chains as either a ketene (*m/z* 500 and 464) or a free acid (*m/z* 482). Further MS/MS analysis of the corresponding [M+Cl^−^]^−^ ion at *m/z* 776.536 revealed the attached acyl linked fatty acids. The prominent ion at *m/z* 277.217 corresponds to a 18∶3 (exact mass 227.2168). Given the presence of this fatty acid, it is likely that this PC, like the pPE discussed above, has an ether-linked chain. The product ions at *m/z* 466.329 and *m/z* 448.319 correspond to loss of this 18∶3 acyl chain as a ketene and free acid respectively. As with the pPE above, the mass of the product ions corresponding to loss of the acyl chains is more consistent with an ether-linked chain than an acyl linked chain. These PC, unlike the pPE were not susceptible to acid hydrolysis (data not shown) making these PCs likely 1-O-alkyl-2-acyl-glycerophosphocholines (aPC) [34]. Using this analysis, however, we cannot definitively determine the location of the acyl or alkyl chains.

Several different aPC molecular species with the acyl chain compositions shown in Table 4 are detected. The aPCs were identified using positive ion MS/MS by the presence of the product ion at *m/z* 184. The negative ion MS/MS spectra of the *m/z* corresponding to [M+Cl^−^]^−^ were analyzed to detect the acyl chain esterified to the PC.

**Table 4:** Alkyl-phosphatidylcholine species identified in *A. pallida* lipid extracts.

[M+H]^+^	Acyl Chain Composition*	Acyl Chains Observed#
740.6	34∶4	16∶1a, 18∶3
742.6	34∶3	16∶0a, 18∶3
744.6	34∶2	16∶0a, 18∶2
746.6	34∶1	16∶0a, 18∶1
754.6	35∶4	17∶1a, 18∶3
756.6	35∶3	17∶0a, 18∶3
758.6	35∶2	17∶0a, 18∶2
760.6	35∶1	17∶0a, 18∶1
766.6	36∶5	16∶0a, 20∶5
768.6	36∶4	16∶0a, 20∶4
		18∶0a, 18∶4
		18∶1a, 18∶3
770.6	36∶3	16∶0a, 20∶3
		18∶0a, 18∶3
772.6	36∶2	18∶0a, 18∶2
774.6	36∶1	18∶0a, 18∶1
780.6	37∶5	17∶0a, 20∶5
782.6	37∶4	17∶0a, 20∶4
		17∶1a, 20∶3
		19∶0a, 18∶4
784.6	37∶3	17∶0a, 20∶3
		19∶0a, 18∶3
792.6		16∶0a, 22∶6
794.6	38∶5	16∶0a, 22∶5
		18∶0a, 20∶5
796.6	38∶4	18∶0a, 20∶4
		18∶1a, 20∶3
798.6	38∶3	18∶0a, 20∶3
800.6	38∶2	18∶0a, 20∶2
806.6	39∶2	17∶0a, 22∶6
		17∶1a, 22∶5
808.6	39∶5	17∶0a, 22∶5
810.6	39∶4	17∶0a, 22∶4
812.6	39∶3	17∶0a, 22∶3
814.6	39∶2	17∶1a. 22∶3
820.6	40∶6	18∶0a, 22∶6
822.6	40∶5	20∶0a, 20∶5
824.6	40∶4	20∶0a, 20∶4
826.6	40∶3	20∶0a, 20∶3
828.6	40∶2	20∶0a, 20∶2
834.6	41∶6	19∶0a, 22∶6
836.6	41∶5	19∶0a, 22∶519∶1a, 22∶4
838.6	41∶4	19∶0a, 22∶4
840.6	41∶3	19∶0a, 22∶3

* Total number of carbons:total unsaturations.

# The alkyl linked acyl chain is indicated by an “a”.

### Phosphonosphingolipids Identified in *A. pallida*

**Figure 7:**
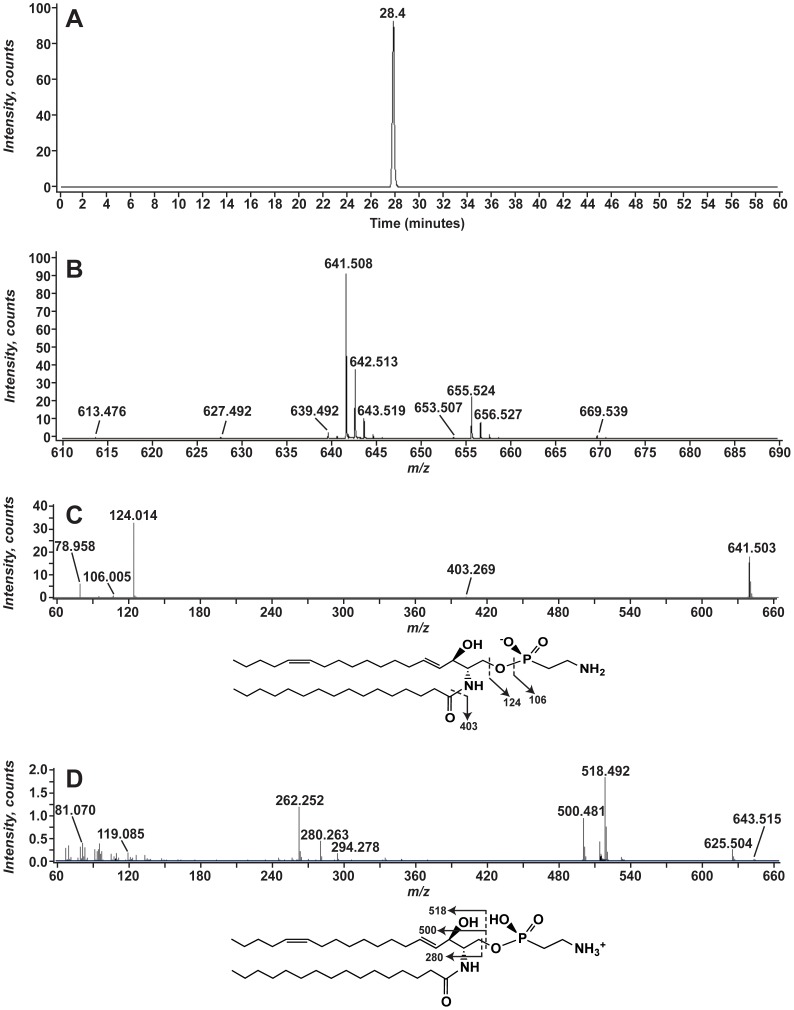
Mass spectrometry of the *A. pallida* phosphonosphingolipid at *m/z* 641.5. Panel A shows the EIC for 641.5. Panel B shows the negative-ion mass spectrum from *m/z* 610 to 690 for the lipids eluting between 27.3 and 28.1 minutes. The MS/MS spectra obtained in the negative-ion mode (Panel C) and the positive-ion mode (Panel D) are shown. The structures below each MS/MS spectra indicate predicted structure as well as selected product ions.

The [M-H]^−^ ion at *m/z* 641.5 was detected in the aposymbiotic and symbiotic anemone lipid extracts (Figure 2, panel A and C). When analyzed by LC-MS, this lipid eluted as a single peak at ∼27.8 minutes (Figure 7A). Several related lipids, at *m/z* 627.492, 639.492, 653.507, 655.524, and 669.539, were also detected and attributable to differences in acylation and unsaturation of a similar lipid species (Figure 7B). MS/MS analysis of the ion at *m/z* 641.508 revealed the spectrum shown in Figure 7C. The product ion at *m/z* 402.269 corresponds to the loss of a 16∶0 fatty acyl chain as a ketene (RCH = CO). There are prominent product ions at *m/z* 124.014 and 78.958. The ion at *m/z* 78.958 is interpreted as PO_3_^−^ and the ion at *m/z* 124.014 corresponds to aminoethyl phosphonate (C_2_H_7_NO_3_P^−^, exact mass 124.0169). MS/MS analysis of the corresponding [M+H]^+^ ion at *m/z* 643.5 yielded the spectra shown in Figure 7D and strongly suggests that this lipid is a phosphonosphingolipid [35]. The ion at *m/z* 625.504 indicates loss of water, likely the 3-hydroxyl of the sphingoid base. The ions at *m/z* 518.492 and 500.381 correspond to loss of the aminoethyl phosphonate from the precursor ion and the dehydrated ion at *m/z* 625 respectively. Likewise, the ions at *m/z* 280.263 and *m/z* 262.252 correspond to loss of the amide-linked chain without and with the 3-hydroxyl loss.

Several different molecular species of this phosphonosphingolipid were identified in aposymbiotic *A. pallida* lipid extracts (Table 5). All of the molecular species were identified by exact mass and revealed similar MS/MS spectra in both the positive and negative ion mode. The fatty acyl chains indicated were identified as product ions in the negative-ion MS/MS analysis of each precursor ion.

**Table 5:** Phosphonosphingolipid species identified in *A. pallida* lipid extracts.

[M-H]^−^	Total carbons and unsaturations*
613.5	34∶2
615.5	34∶1
627.5	35∶2
629.6	35∶1
639.5	36∶3
641.5	36∶2
653.5	37∶3
655.5	37∶2
669.5	38∶2
671.5	38∶1

* In the ceramide portion.

### Sulfoquinovosyldiacylglycerol Identified in *Symbiodinium* FLAp1AB

**Figure 8:**
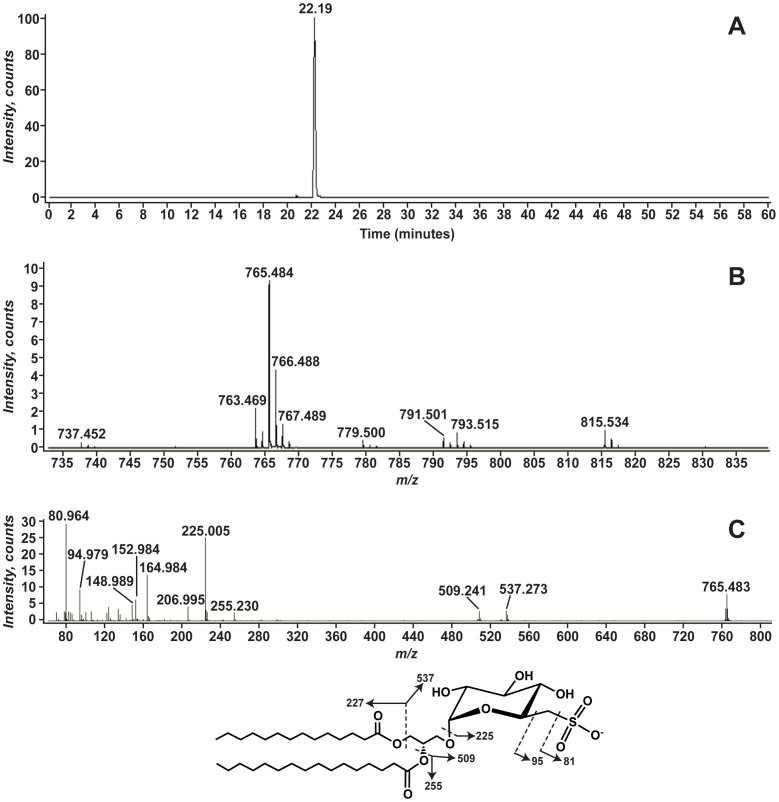
Negative-ion mass spectrometry of the *Symbiodinium* sulfolipid at *m/z* 765.5. Panel A shows the EIC for [M-H]^−^ ion at *m/z* 765.5. Panel B shows the mass spectrum from *m/z* 735 to 835 for the lipids between 22.0 and 22.4 minutes. Panel C shows the MS/MS spectrum of *m/z* 765.5. The predicted structure as well as selected product ions is shown beneath the spectrum.

The [M-H]^−^ ion at *m/z* 765.484 is derived from *Symbiodinium* and appears as a prominent ion in symbiotic anemone lipid extracts (Figure 2). During LC-MS analysis, this lipid elutes as a single peak at a retention time of ∼22 minutes (Figure 8A). Eluting with it are several other ions with *m/z* 737.452, 763.469, 779.500, 791.501, and 793.515 that are related to 765.5 by differences in acyl chain composition (Figure 8B). MS/MS analysis of the ion at *m/z* 765.5 (Figure 8C) reveals ions that are consistent with an acylated lipid. The product ions at *m/z* 537.273 and 509.241 correspond to the mass expected for the loss of a 14∶0 and 16∶0, respectively, where the acyl chains are lost as free fatty acids (RCOOH). In addition, product ions corresponding to those fatty acids are detected at *m/z* 227.204 (14∶0, exact mass: 227.2044) and 255.235 (16∶0, exact mass: 255.2324). A prominent product ion at *m/z* 80.964 is interpreted as sulfonate, SO_3_H^−^ (exact mass: 80.9652) [36]. An additional product ion at *m/z* 225.010 is consistent with sulfoquinovosyl (C_6_H_9_O_7_S^−^, exact mass 225.0074) [35], [37]. Taken together this strongly suggests that the lipid at *m/z* 765.484 is a sulfoquinovosyldiacylglycerol (SQDG).

A variety of SQDG molecular species were identified in the *Symbiodinium* lipid extracts (Table 6). All yielded, upon negative ion MS/MS analysis, product ions corresponding to fatty acid loss, sulfonate, and sulfoquinovosyl consistent with their exact mass.

**Table 6:** SQDG species identified in FLAp1AB lipid extracts.

[M-H]^−^	Acyl Chain Composition*	Acyl Chains Observed
735.5	28∶1	12∶0, 14∶1
737.5	28∶0	12∶0, 14∶0
763.5	30∶1	14∶0, 16∶1
765.5	30∶0	14∶0, 16∶0
791.5	32∶1	16∶0, 16∶1
793.5	32∶0	16∶0, 16∶0
817.5	34∶1	16∶1, 18∶1
819.5	34∶1	16∶0, 18∶1
821.5	34∶0	16∶0, 18∶0

* Total number of carbons:total unsaturations.

### XCMS Comparisons of the Lipid Compositions of Symbiotic and Aposymbiotic Anemones

In order to identify changes in the host and symbiont lipidomes that occur when the partners are living in symbiosis, we attempted to do a subtractive analysis to compare the total negative-ion LC-MS data. Symbiotic and aposymbiotic anemones were harvested three times over seven days and used to generate triplicate lipid extracts from ∼0.1 g of animal tissue. These lipid extracts were analyzed using negative-ion LC-MS and the data was then aligned, normalized and compared using the Scripps Center for Metabolomics XCMS online resource [27], [38].

**Figure 9:**
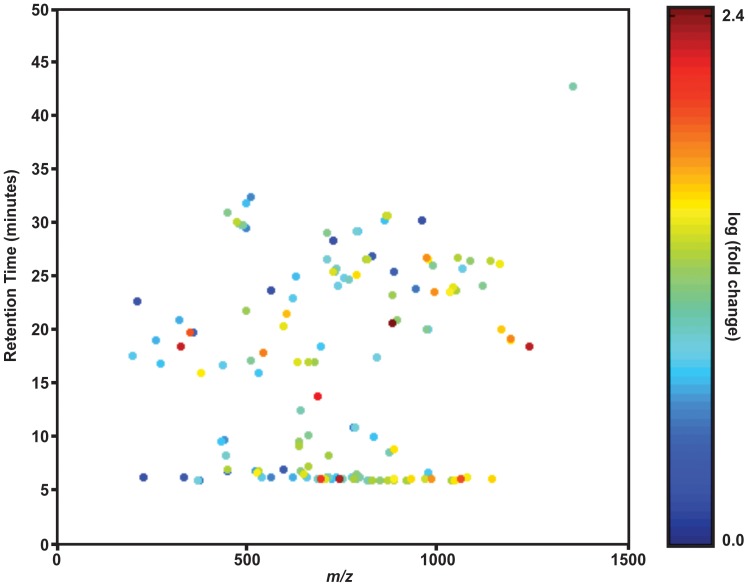
XCMS comparison of the lipid profiles of aposymbiotic vs. symbiotic *A. pallida*. Biological and technical triplicates of aposymbiotic and symbiotic anemones (∼0.10 g each) were analyzed using negative-ion normal phase LC-MS. The spectra were aligned, normalized and compared using XCMS [27], [38]. Each circle on the plot represents a lipid species as identified by *m/z* and retention time. The color of the circle indicates the fold change between the aposymbiotic and symbiotic data sets. The plot shows the features, after isotopes and *Symbiodinium* features were removed that differed between the aposymbiotic and symbiotic samples with p<0.0001. The fold change indicates that the feature is higher in aposymbiotic anemones as compared to symbiotic anemones or vice versa. The entire data sets are available in the supporting information.

Of the 18543 distinct features identified by retention time and *m/z* in these data, 447 were considered to be statistically different between the aposymbiotic and symbiotic anemones (p<0.0001). Of these, we subtracted lipid features that were derived from the symbiont as well as features that corresponded to isotopes of identified lipids leaving 255 features. Each of the EICs for the remaining features were screened to remove any features that did not yield an EIC above the noise of the sample (see Figure S1 for an example). Figure 9 shows the 149 features that differed between aposymbiotic and symbiotic *A. pallida* (p<0.0001). Table S1 shows the entire and truncated feature lists.

**Figure 10:**
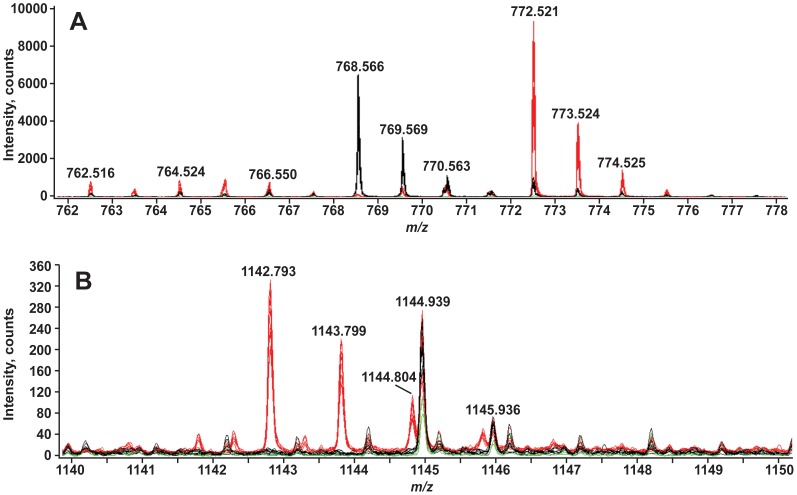
Lipids that differ between aposymbiotic and symbiotic lipid extracts. The figures show the overlay of the negative-ion LC-MS of 9 aposymbiotic (black), 9 symbiotic (red) and 3 FLAp1AB (green) lipid extracts. Panel A shows the lipids from *m/z* 710 to 805 that elute between 23.8 and 24.2 minutes. Based on MS and MS/MS we hypothesize that the lipid at *m/z* 772.5 is 39∶2 pPE and at *m/z* 768.5 is 39∶4 pPE. Panel B shows the lipids from *m/z* 1140 to 1150 that elute between 25.9 and 26.6 minutes. The exact structure of the lipid at *m/z* 1142.8 has not been determined.

Two examples of lipids that changed significantly are shown in Figure 10. Panel A illustrates changes in the [M-H]^−^ ions at *m/z* 768.5 and 772.5 between the aposymbiotic and symbiotic anemones. These ions are hypothesized, by exact mass and MS/MS, to be 39∶4 and 39∶2 pPE. The peak area of the EIC of the 768.5 ion is 90 fold higher in the aposymbiotic anemone as compared to the symbiotic anemone. Conversely, in the symbiotic anemone the ion at *m/z* 772.5 is ∼10 fold higher than in the aposymbiotic anemone. This data illustrates that the relative abundance of lipids both increase and decrease between the symbiotic and non-symbiotic states. Furthermore, this clearly suggests that there is remodeling of acyl chain composition in anemone-derived lipids to accommodate symbiosis.

Panel B illustrates changes in an unidentified lipid at *m/z* 1142. 8. This lipid is identified as an [M-H]^−^ ion and is likely related to an [M-H]^−^ ion at *m/z* 1170.8. The additional 28 mass units can be interpreted as an increase in acyl chain length by two carbons. Both of these features differ significantly between the aposymbiotic and symbiotic anemones. The ions at *m/z* 1142.8 and 1170.8 are ∼21 and ∼31 fold, respectively, more abundant in symbiotic anemones than in aposymbiotic anemones. MS/MS analysis of these species did not reveal a fragmentation pattern that matched any lipid species known to these researchers.

## Discussion

In this work we report preliminary characterization of the lipidome of the model anemone *A. pallida* living both in and out of symbiosis with its dinoflagellate symbiont *Symbiodinium*. High-resolution accurate-mass quadrupole time-of-flight mass spectrometry of the total lipid extracts from symbiotic anemones, aposymbiotic anemones and *Symbiodinium* reveals the complexity of these under-characterized lipidomes. Previously, thin layer chromatography identified PE, PS and PC in the symbiotic anemone *Anthopleura elegantissima* [39]. Here we have identified typical 1, 2-diacyl GPLs, such as PI and PS, as well as 1-alkyl-2-acyl GPL forms of PC and PE. The acid sensitivity of the PE pool strongly indicates that it is a plasmalogen form of PE [25]. The presence of GPLs with alkyl linked acyl chains has not been previously observed and furthers our knowledge of the lipid composition of anemones such as *A. pallida*. In addition, through MS and MS/MS analysis we have been able to identify the molecular diversity of these GPLs, including the acyl chain composition. This data corroborates data on the fatty acid composition previously shown 15,40,41 but expands upon this previous work by associating fatty acids with these various classes of polar lipids. Future work analyzing the total fatty acid composition of this system is warranted as our analysis cannot distinguish among the many possible isobaric fatty acids [15], [40], [41].

By comparing multiple biological and technical replicates, we gained insights into the comparative changes that occur between the aposymbiotic and symbiotic states. We observed lipids that were increased in the aposymbiotic anemone relative to the symbiotic anemone and vice versa. While we report only on a subset of the alterations to the lipidome, the data we have generated can be used to fuel further research of the diverse changes to the lipidome of *A. pallida* in and out of symbiosis with its symbiont *Symbiodinium*. For example, the molecular composition of 39∶2 pPE is present at ∼10 fold higher levels in symbiotic as compared to aposymbiotic anemones. The 39∶4 pPE, for example, is ∼90 fold higher in aposymbiotic anemones as compared to symbiotic anemones. This result may suggest that acyl-chain remodeling is occurring to the pPE pool as symbiosis is established or maintained. The fatty acids may be released for use as fuel or for the synthesis of signaling lipids. Further investigation of the acyl-chain composition of the pPE and the other GPLs is clearly warranted based on this result.

Statistically, this 39∶4 pPE feature did not meet our stringent cut-off for inclusion in the comparative profile shown in Figure 9 (p<0.0001) though it did meet the FDR-adjusted cut-off (FDR cut-off of 0.01, p = 8.3×10^−4^). Further screening of the 1539 lipids that met the above criteria will likely give insights into the lipidomic changes occurring during symbiosis.

A number of lipid features that were statistically different between the aposymbiotic and symbiotic anemones eluted between 6–7 minutes off the normal phase LC column. These lipids are likely non-polar lipids, such as tri- and di-acylglycerols, and sterols [15], [42] that are not effectively retained by the column. The large number of these lipids that met our stringent criteria suggests that this class of lipids plays an important role in symbiosis. This may point to an increased dependence on lipids for energy or signaling. These lipids, which remain to be identified, maybe more effectively analyzed using reverse phase LC prior to MS analysis or analysis in the positive-ion mode.

The lipid composition of *Symbiodinium* was largely not characterized in this study due to our inability to match the MS and MS/MS data we collected with lipid structures known to these researchers. One lipid clearly identified by exact mass and MS/MS was SQDG. This sulfolipid is often found in photosynthetic organisms [43] such as *Symbiodinium*. It has been detected in plants [43], [44], marine algae [45], eubacteria [46], archaebacteria [46], *Rhizobiaceae* [47], and *Bacillus* [48]. SQDG has also been found associated with photosystem II [49], and is proposed to play a role in chloroplast development [50], [51]. The fatty acid composition of SQDG observed in *Symbiodinium* FLAp1AB mirrors that reported previously [52]; 16∶0 and 18∶1 were significantly represented in the SQDGs observed.

SQDG has also been implicated in plant response to phosphate (P_i_) starvation. As P_i_ becomes depleted, GPLs are replaced with non-phosphate containing lipids such as monogalactosyldiacylglycerol (MGDG), digalactosyldiacylglycerol (DGDG), and SQDG [43], [44]. SQDG may play an important role in this process; as an anionic lipid it may help replace loss of the anionic GPL phosphatidylglycerol (PG) [43].

The detection of SQDG in total lipid extracts of symbiotic anemones (Figure 2) suggests that its levels maybe relatively increased compared to the non-symbiotic FLAp1AB. Given that the symbiont biomass comprises a small fraction of the total biomass of a symbiotic anemone, this is of interest because of the role that this lipid has been found to play in response to temperature changes and other environmental disturbances in plants [53]. Perhaps upon entry into symbiosis, the levels of this lipid increase in the chloroplast to adjust to changes in the environmental milieu represented by the host cell.

Recently, Awai *et al* reported that *Symbiodinium* the lipid and fatty acid composition of *Symbiodinium* [54]. Using TLC co-migration with standards, they proposed that *Symbiodinium* possess PE, PS, PG, phosphatidic acid (PA), cardiolipin, SQDG, MGDG, and DGDG. Using normal phase LC elution time, MS and MS/MS we detected PG in *Symbiodinium* (data not shown) but no other common GPLs. As GPLs such as PS, PC, PE, and PA give characteristic MS/MS product ions derived from the glycerol phosphate moiety [32] that is easily identified in the negative-ion mode, this calls into question the results of Awai *et al*. Taken together, more careful analysis of the *Symbiodinium* lipid composition is warranted.

Phosphonosphingolipids were also identified in *A. pallida*. These unusual lipids are widely observed in marine invertebrates [55] and have been observed in anemones previously [39], [56]. The presence of the C-P bond in the aminoethyl phosphonate head group is resistant to hydrolytic enzymes [57], [58]. This may indicate an important role for this lipid in the establishment of symbiosis. The stability of these phosphonosphingolipids may help maintain cellular integrity as the organism is engulfed into the gastrodermal cells and the symbiosome membrane is generated. The identification of this class of lipids also opens the possibility that other phosphonolipids such as phosphonosphingoglycolipids [56] may be present in this system.

We show in this study that the establishment of symbiosis in cnidarians is accompanied by significant changes in the lipidome of the anemone host. In this study we have made preliminary efforts to identify lipids present in this system and have identified some of the changes that occur. Much work needs to be done to comprehensively characterize these complex lipidomes. We have not yet identified MGDG and DGDG though they are extremely likely to be present in this symbiotic pair. In addition, this MS-based analysis could be carried out detecting in the positive-ion mode to facilitate identification of other lipids. Future work should focus on relating the lipidomic changes with the establishment of the symbiosome membrane, remodeling of the plasma membrane, the signaling roles they may play in communicating between host and symbiont, and the energetic roles that they play in the metabolism of the partners.

## Supporting Information

Figure S1 **Examples of EICs that were used for screening the feature list.** Panel A shows an example of an EIC that was judged to not be above the noise of the sample and therefore removed from the list. Panel B shows the EIC of a feature that was retained on the list.
(PDF)

Table S1 The feature lists generated using XCMS. The truncated and un-truncated data sets are shown. For each data set the file is divided into three sections. In the first six columns, the fold change between the aposymbiotic and symbiotic anemone lipid extracts (fold), average *m/z* (*m/z*), average retention time, and p-value for each feature is shown. The mean peak area and standard deviation for the aposymbiotic and symbiotic data are shown in the next four columns. The last eighteen columns show the peak areas of the extracted ion current for each feature for each replicate as determined by XCMS. For the truncated data set the asterisk indicates that a feature was higher in the aposymbiotic state versus the symbiotic state.
(XLSX)
