# Supplementary figures and images for: Comparative Lipid Profiling of the Cnidarian Aiptasia pallida and Its Dinoflagellate Symbiont
Source: PLoS One. 2013 Mar 4;8(3):e57975. doi: 10.1371/journal.pone.0057975 (PMC3587569; doi:10.1371/journal.pone.0057975)

Figure S1

**A**

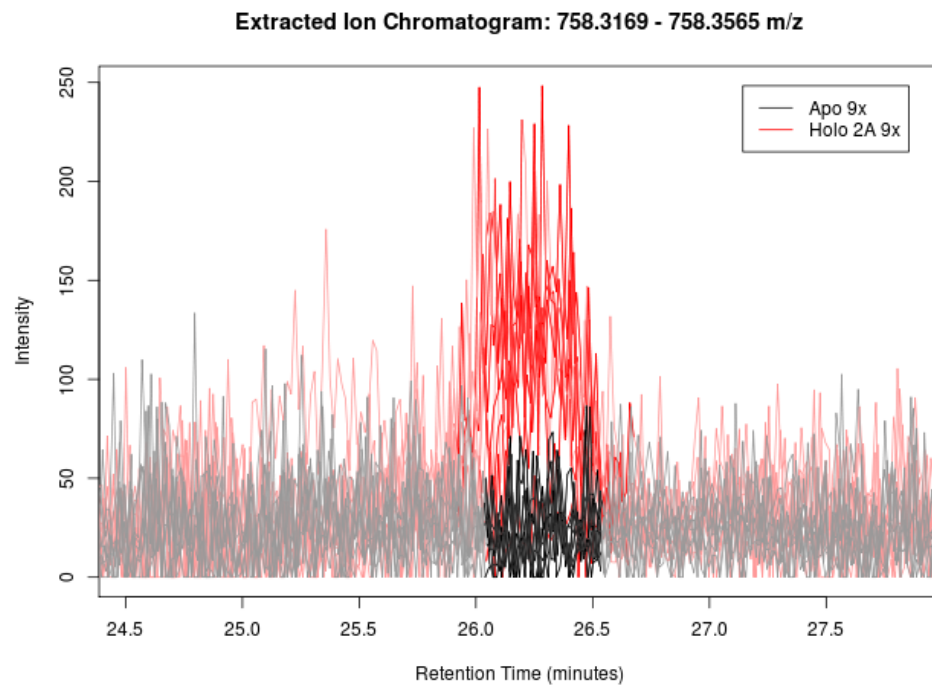

**B**

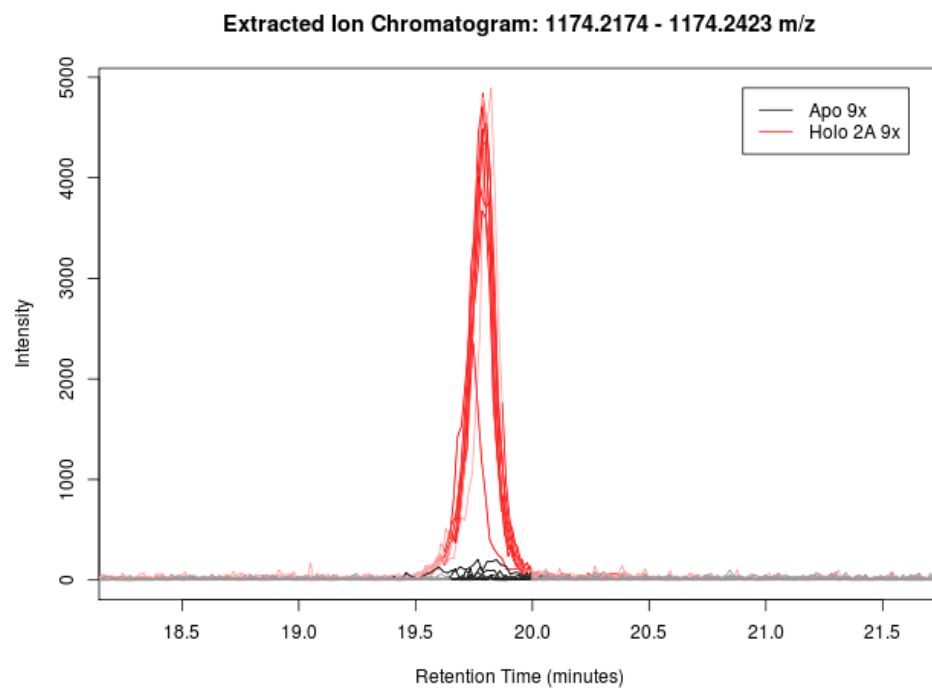

Supplement: Figure S1 — Examples of EICs that were used for screening the feature list. Panel A shows an example of an EIC that was judged to not be above the noise of the sample and therefore removed from the list. Panel B shows the EIC of a feature that was retained on the list. (PDF) [file pone.0057975.s001.pdf]
